# Supplementary material for: HIVprotI: an integrated web based platform for prediction and design of HIV proteins inhibitors
Source: J Cheminform. 2018 Mar 9;10:12. doi: 10.1186/s13321-018-0266-y (PMC5845081; doi:10.1186/s13321-018-0266-y)
Supplement: Supplementary file 2 — Additional file 2. Source code of HIVProtI web server. [file 13321_2018_266_MOESM2_ESM.zip › HIVprotI_Source-code/hivprotI/weka-3-6-6/doc/weka/core/Optimization.html]

Optimization


---


|  |  |  |
| --- | --- | --- |
| **Overview** \| **Package** \| **Class** \| **Tree** \| **Deprecated** \| **Index** \| **Help** \| **Weka's home** | |  |
| **PREV CLASS**   **NEXT CLASS** | **FRAMES**    **NO FRAMES**     **All Classes** |  |
| SUMMARY: NESTED \| FIELD \| CONSTR \| METHOD | DETAIL: FIELD \| CONSTR \| METHOD |  |


---


## weka.core Class Optimization

```
java.lang.Object
  weka.core.Optimization
```

**All Implemented Interfaces:**: RevisionHandler, TechnicalInformationHandler

---

``` public abstract class Optimization extends java.lang.Object implements TechnicalInformationHandler, RevisionHandler ```

Implementation of Active-sets method with BFGS update to solve optimization
problem with only bounds constraints in multi-dimensions. In this
implementation we consider both the lower and higher bound constraints.

Here is the sketch of our searching strategy, and the detailed description
of the algorithm can be found in the Appendix of Xin Xu's MSc thesis:

Initialize everything, incl. initial value, direction, etc.

LOOP (main algorithm):
